# An Analysis of the Nutritional Value of Diets Generated by Large Language Models Under Average User Conditions—3(LM)Diet Study

**DOI:** 10.3390/nu18142363

**Published:** 2026-07-18

**Authors:** Hubert Dobrowolski

**Affiliations:** School of Medical & Health Sciences, VIZJA University, Okopowa 59 Str., 01-043 Warsaw, Poland; h.dobrowolski@vizja.pl

**Keywords:** LLMs, AI, ChatGPT, Gemini, Copilot, diet, nutritional standards compatibility, nutritional value, generated diet

## Abstract

**Background/Objectives**: AI tools are becoming increasingly common, including in areas related to health and diet. However, there are no studies indicating the nutritional value of diets generated by large language models (LLMs). The aim of this study was to analyse the nutritional value of diets generated by three major language models. **Methods**: Three LLMs were used in the study: ChatGPT, Gemini and Copilot. Using each of these, 35-day meal plans were generated for three energy variants: 2000 kcal, 2500 kcal and 3000 kcal. The meal plans were then analysed using dietary software. **Results**: All LLMs generated meal plans with an insufficient energy value (*p* < 0.001, one-sample *t*-test) by 284–546 kcal, depending on the model and the targeted energy content. The meal plans were characterised by an excessively high proportion of energy from protein and an excessively low proportion of energy from fat. Carbohydrates were planned in the correct amounts. ChatGPT and Copilot generated meal plans with a generally adequate vitamin and mineral content. Gemini generated meal plans that were often deficient, particularly for diets with lower energy values. **Conclusions**: Diets generated by LLMs have many shortcomings. Unsupervised use of LLM-generated meal plans by non-expert users may produce nutritionally inaccurate diets and should not replace professional dietary counselling.

## 1. Introduction

Diet is undoubtedly one of the key factors influencing an individual’s health. A diet with an appropriately calculated energy intake allows for effective weight management, whilst adequate intake of macronutrients and micronutrients helps maintain optimal bodily functions and plays a role in the prevention and treatment of many diseases [1,2,3]. Although in recent years particular attention has been paid to the quality of the diet, the intake of specific nutrients has been linked to many diet-related diseases and is certainly crucial to maintaining good health [4,5,6,7,8,9,10]. The impact of the intake of selected nutrients is therefore often reflected in numerous dietary recommendations [11,12,13,14]. An adequate intake of nutrients is therefore crucial for both individual and public health.

Growing public awareness means that many people are trying to maintain a healthy diet. However, research suggests that the general public’s knowledge of nutrition is often inadequate, highlighting the need for further education [15,16,17,18,19]. Many people therefore try to find the right information, often using unsuitable sources. One of the most popular sources of knowledge is the internet [18,20]. Despite its many undeniable advantages and often reliable nutritional information, the internet is also rife with numerous misconceptions and myths that are difficult for the average user to spot and can be misleading [21]. A lack of nutritional knowledge may prevent users from properly verifying the information obtained, which in turn may result in an inadequately compiled diet.

In recent years, AI tools—particularly large language models (LLMs)—have become increasingly popular as sources of information [22]. LLMs are a class of machine learning models designed to process, understand and generate human language, and they are capable of learning from a wide variety of linguistic data through self-supervised learning [23]. In practice, working with an LLM is usually similar to working with a search assistant, where the user does not typically ‘hand over a task’ but instead iterates; they formulate a prompt, clarify their requirements, refine the response and ask for another version until the desired result is achieved [24]. Prompts are a series of instructions designed to elicit the desired responses from an LLM, and formulating them correctly is crucial for establishing the right context and obtaining high-quality responses [24,25]. Well-formulated prompts, along with appropriate materials and statements provided to the LLM, can result in reliable knowledge and well-formulated answers. However, the average user often lacks the knowledge and skills to use these platforms effectively. Inappropriately formulated queries and prompts can result in information of dubious accuracy, which, in the field of medicine and health, can be dangerous, as research confirms [26,27]. The inappropriate use of LLMs can also pose a serious risk when it comes to dietary advice and suggestions for specific food items on menus. A case has been reported of serious consequences resulting from the uncritical application of advice provided by AI and LLMs regarding diet [28].

Research into the ability of LLMs to generate dietary plans and their potential health implications remains limited. To date, much of the research has focused on the use of these models for nutritional advice [29,30,31] or their potential in disease management [32,33,34,35]. Some studies have also focused on the ability to generate diets in terms of energy content and nutrient composition for diets of varying energy values [36], as well as in the context of diabetes [35] and obesity [37]. However, these studies involved diets with a very short duration (1–5 days) and generally focused on a single LLM. One study used the three most popular models to generate 10-day meal plans; however, it compared only the quality of the diet, and the recommendations provided focused solely on a specific case and calorie intake [38]. Consequently, there is a lack of studies assessing the usefulness of meal plans over a longer period of time, as well as in specific conditions unique to a given country, in terms of current dietary guidelines, current needs, or economic conditions.

The aim of the 3(LM) Diet project is to investigate the potential of LLMs to formulate diets within a broader, multidimensional framework. The study covers the main areas of interest to contemporary society regarding the characteristics a diet should possess—namely, health, economic and environmental aspects. The aim of this study was to demonstrate these capabilities when the models are used by a user who lacks the necessary knowledge to formulate queries correctly, provide relevant data, and verify the accuracy of the responses received. The main objective is to analyse the risks associated with the use of language models by such users and, subsequently, the potential consequences.

Given the growing popularity of language models, the risks arising from their misuse, and the crucial role of diet in maintaining health both at the individual and population levels, the aim of this study was to analyse the nutritional value of diets generated by three major language models and the potential adverse health effects of these diets.

## 2. Materials and Methods

Three models were used to generate the menus: ChatGPT (GPT-5.5, accessed 11 May 2026) [39], Gemini (Gemini 2.5 Flash, accessed 12 May 2026) [40], and Microsoft 365 Copilot (based on the GPT-5 model, accessed 15 May 2026) [41]. All prompts were executed between 11 May 2026 and 25 May 2026. No intentional model updates were introduced by the investigators during data collection. These models were selected due to their immense popularity among users, their ease of access, and the high public recognition of the brands behind these tools. To generate the meal plans, free accounts were used that had not previously generated information related to diet or a healthy lifestyle in order to avoid conversation history or personalisation effects using these tools. Free accounts made it possible to simulate users with an amateurish approach who were more likely to be unfamiliar with the correct methods of creating prompts. All generations were performed in Warsaw, Poland.

In the initial task, all models were given the same instruction to generate a 35-day meal plan. All commands were issued in Polish in order to accurately replicate the circumstances of the average user in a given country, who may not necessarily have a sufficient proficiency in a foreign language. All guidelines—including energy content, compliance with nutritional recommendations and standards, and additional aspects of the meal plans—were carefully outlined. The meal plans were to be balanced on a weekly basis, taking into account the user’s possible preferences regarding the order of individual weeks; however, the entire 35-day dietary plan should ultimately also be balanced. Each LLM was to generate, in separate commands, a 2000 kcal, 2500 kcal, and 3000 kcal diet. Each model, therefore, generated 105 days of meal plans with varying energy values. In cases where the correct number of days was not generated (e.g., generating 5 days instead of 5 weeks), or where the first few days were generated in one format and subsequent days in an abbreviated version (e.g., presenting 2 weeks, followed by a recommendation to mix individual meals), the model was asked to correct this by presenting a realistic 35-day plan in order to avoid randomness regarding the fulfilment of requirements for specific nutrients. Once generated, all menus were evaluated by a dietitian. The LLMs were then asked to clarify the weights and/or recipes of the products and dishes proposed in the menus, but only until it became absolutely necessary for determining the nutritional values of the meals and diets generated. No modifications intended to improve the nutritional quality of the meal plans were introduced. Any uncertainties regarding the products were resolved by a dietitian, considering the Polish context and the conditions set out in the prompt. This resulted in menus that left no doubt as to their exact composition and could be assessed unambiguously. Due to varying shortcomings, the models were given individual instructions, though using the same phrasing, such as ‘specify’, ‘provide’, ‘present’, etc. The commands were therefore as similar as possible across the LLMs. Ultimately, a maximum of 4 additional commands were used in the models. Only these refined menus were subsequently assessed. The entire procedure is presented in Table 1. The exact prompts, all files from Dieta 6.0 software, and generated data can be found in the open data repository (https://doi.org/10.18150/9GVUEG).

In addition to providing an appropriate energy intake, the diet had to meet additional guidelines. It had to be designed for a healthy individual with no dietary restrictions and a normal body weight so as not to restrict participants in their choice of products and dishes nor impose specific recommendations, including a negative energy balance. The menu was to comply with Polish dietary recommendations [11,42] and nutritional standards [43]. In addition, it was intended for people aged 19–30 years. This age range is determined by the popularity of language models among people of a similar age, as well as the age range of the population groups for which dietary guidelines for specific nutrients have been established [43]. The diet was designed to meet the criteria of a balanced, environmentally friendly diet—a key challenge of the modern world [1,44]—and to be affordable within the Polish context, in line with data on average food expenditure in the most recent calendar year reported by the Central Statistical Office [45]. Meal plans were therefore designed to suit the average Polish resident, who might use LLM to generate a diet plan.

The generated diets were checked for completeness of data, nutritional composition and weights. Subsequently, all meal plans were analysed using the Dieta 6.0 software (NIZP-PZH, Warsaw, Poland). This software is a professional dietary software published by the National Institute of Public Health—National Institute of Hygiene, which is responsible for the latest edition of Polish dietary recommendations and standards. The software also includes products and dishes available on the Polish market, the compositions of which are taken from the most up-to-date tables of food composition and nutritional values applicable in Poland [46]. The generated diets were analysed in terms of energy value, macronutrient composition, and the content of specific vitamins and minerals and then compared with the current nutritional standards for the Polish population [43]. The EAR standard was selected as the default. Where no EAR standard had been specified, the AI standard was applied. Given the different recommended values for vitamins and minerals for each gender, meeting nutritional standards for these nutrients was determined separately for both women and men. In assessing the energy values, consideration was given to whether the plans achieved the exact target energy value or fell within a possible 10% deviation from that target.

Statistical analysis was performed using IBM SPSS Statistics v. 31.0.1.0 (IBM Corp., Armonk, NY, USA). The Shapiro–Wilk test was used to assess the normality of the distribution. Descriptive statistics were used to present the energy and nutritional values. To assess the agreement between the generated energy value and the desired value, the one-sample *t*-test was used for normally distributed variables and the one-sample Wilcoxon test for variables with a non-normal distribution. The Kruskal–Wallis test was used to compare differences between the actual and expected energy values of the diets across the individual LLMs. In the post hoc analysis, the Dunn test with Bonferroni correction was used. ANOVA was used to compare the amounts of macronutrients provided by the menus across the individual LLMs, where the assumption of homogeneity of variances was met (for protein). The Scheffe test was used to examine pairwise relationships as part of the post hoc analysis. Where the assumption of homogeneity of variances (for fat and carbohydrates) was not met, the Welch test was applied. In this case, the Tamhane test was used for the post hoc analysis. The vitamin and mineral content in the generated menus was presented in quantitative terms and as a percentage of menus falling below nutritional standards. The Kruskal–Wallis test was used to compare the content of individual minerals and vitamins among the LLMs. As with the analysis of differences in energy values, the post hoc analysis used the Dunn test with Bonferroni correction. The analyses were carried out only between models. No comparisons were made between individual values within a given model. Statistical significance was set at α = 0.05.

## 3. Results

### 3.1. Energy Value

The energy values of the generated meal plans are presented in Table 2. The meal plans generally had a lower energy value than that specified in the instructions. All language models generated significantly lower energy values than the target value (*p* < 0.001, *t*-test and Wilcoxon test). This applied to all models across all three energy targets.

When attempting to generate 2000 kcal diets, the mean value generated by ChatGPT was 1711 ± 169 kcal/day, whilst the median energy values for Gemini and Copilot were 1540 kcal/day and 1699 kcal/day, respectively. The energy value of the meal plans generated by Gemini was significantly lower than the energy values generated by ChatGPT and Copilot (*p* < 0.001, Kruskal–Wallis test). No statistically significant differences were observed between ChatGPT and Copilot (*p* = 0.296, Kruskal–Wallis test). Only 2.9% (n = 1) of the meal plans generated by ChatGPT and 8.6% (n = 3) of those generated by Copilot achieved the expected energy value. None of the meal plans generated by the Gemini model reached the expected value. Nine (25.7%) meal plans for ChatGPT and 10 (28.6%) meal plans for Copilot fell within a 10% margin of error of the expected value. None of the menus generated by Gemini fell within the 10% margin of error of the expected value.

When attempting to generate meal plans with a target of 2500 kcal, the mean value generated by Gemini was 1965 ± 184 kcal/day, whilst for ChatGPT it was 2152 ± 191 kcal/day; the median energy value generated by Copilot was 2216 kcal/day. The energy value of the meal plans generated by Gemini was significantly lower than the energy values generated by ChatGPT and Copilot (*p* < 0.001, Kruskal–Wallis test). No statistically significant differences were observed between ChatGPT and Copilot (*p* = 0.194, Kruskal–Wallis test). One (2.9%) meal plan generated by ChatGPT met the target value, and 12 (34.3%) meal plans fell within a margin of error of 10%. In the case of Gemini, no meal plan reached the target value of 2500 kcal, whilst 2 (5.7%) differed from this value by less than 10%. Three (8.6%) of the meal plans generated by Copilot reached the value of 2500 kcal/day, whilst 11 (31.4%) fell within the 10% margin of error.

The median energy value of the meal plans generated by Copilot, based on a target of 3000 kcal, was 2454 kcal/day. Two meal plans (5.7%) reached the 3000 kcal target, whilst six meal plans (17.1%) differed by a maximum of 10% from the target value. The average energy value of the meal plans generated by ChatGPT and Gemini was 2522 ± 212 kcal/day and 2629 ± 258 kcal/day, respectively. Two (5.7%) menus generated by Gemini reached 3000 kcal, whilst 13 menus (37.1%) differed from the target value by a maximum of 10%. In the case of ChatGPT, no meal plan reached 3000 kcal, whilst 8 of them (22.9%) differed from the target value by a maximum of 10%. No statistically significant differences were observed between the energy values generated by the language models for diets with a target energy value of 3000 kcal (*p* = 0.153, Kruskal–Wallis test).

Taking into account all the meal plans generated, the difference between the energy value of the meal plans generated by Gemini and the target energy value was significantly greater than in the case of ChatGPT and Copilot (*p* < 0.001, Kruskal–Wallis test). No such differences were observed when comparing the ChatGPT and Copilot models (*p* = 0.301, Kruskal–Wallis test). For meal plans with a target energy value of 2000 kcal, the Gemini model generated diets with a greater deviation from the expected value compared to those generated by ChatGPT and Copilot (*p* < 0.001, Kruskal–Wallis test). No differences were observed between the ChatGPT and Copilot models for diets with this target energy value (*p* > 0.05, Kruskal–Wallis test). Similarly, for meal plans with an energy value of 2500 kcal, the Gemini model generated diets with a greater deviation from the expected value compared to those generated by ChatGPT and Copilot (*p* < 0.001, Kruskal–Wallis test). For this energy value, the difference between the target energy value and that generated by Copilot was significantly smaller than the difference between the target energy value and the diets generated by ChatGPT (*p* = 0.031, Kruskal–Wallis test). For meal plans with a target energy value of 3000 kcal, Gemini generated meal plans with a significantly smaller deviation from the target value than was the case with the Copilot model (*p* = 0.029, Kruskal–Wallis test). No differences were observed between the Gemini and ChatGPT models for diets with an energy value of 3000 kcal (*p* > 0.05, Kruskal–Wallis test).

### 3.2. Percentage of Protein, Fat and Carbohydrates

The diets generated by ChatGPT provided, on average, 22.3 ± 2.5% of energy from protein, 21.8 ± 4.0% from fat and 59.3 ± 4.6% from carbohydrates. The diets generated by Gemini provided 20.6 ± 3.4%, 28.1 ± 3.6% and 53.8 ± 4.7% of energy from protein, fat and carbohydrates, respectively. In the case of Copilot, energy was derived from protein, fat and carbohydrates in amounts of 20.2 ± 2.0%, 26.1 ± 4.3% and 56.8 ± 3.8%, respectively. A statistically significant difference was demonstrated across all energy levels combined between the percentage of energy provided by individual macronutrients across the different language models (*p* < 0.001, ANOVA and Welch test). Post hoc analysis showed that ChatGPT provided a higher percentage of energy from protein compared to the other language models (*p* < 0.001, Scheffe test) and a significantly lower percentage of energy from fat compared to the other models (*p* < 0.001, Tamhane test). Meal plans generated by ChatGPT also provided significantly more energy from carbohydrates compared to Gemini (*p* < 0.001, Tamhane test) but not Copilot (*p* = 0.678, Tamhane test). The menus generated by Copilot provided significantly more energy from carbohydrates compared to those generated by Gemini (*p* < 0.001, Tamhane test). The percentage distribution of macronutrients in the energy content is shown in Table 3.

### 3.3. Vitamins and Minerals

The content of individual minerals in the generated menus and a comparison with the current nutritional standards in Poland are presented in Table 4. The models generated menus which, over the course of individual days, generally met the requirements for sodium, phosphorus, iron, zinc, copper and manganese, and only a small percentage of days failed to meet the standard. Furthermore, the meal plans generated by ChatGPT contained adequate levels of calcium, magnesium and iodine. Similarly, the meal plans generated by Copilot contained adequate levels of magnesium. Most frequently, the meal plans generated by Gemini, particularly those with an expected value of 2000 kcal, contained insufficient amounts of minerals.

Overall, the meal plans generated by Gemini provided significantly less potassium, calcium, iron, zinc and manganese compared with the other models (*p* < 0.05, Kruskal–Wallis test), as well as significantly less sodium, phosphorus, magnesium and iodine compared to ChatGPT (*p* < 0.05, Kruskal–Wallis test) and significantly less copper compared to the Copilot meal plans (*p* = 0.002, Kruskal–Wallis test). Furthermore, the Copilot meal plans contained significantly less potassium, phosphorus, zinc, manganese and iodine than the ChatGPT meal plans (*p* < 0.05, Kruskal–Wallis test). No other differences in mineral content were found between the various LLMs (*p* > 0.05, Kruskal–Wallis test).

The vitamin content of the generated meal plans and a comparison with the current nutritional standards in Poland are presented in Table 5. The menus generated by all language models over the course of individual days generally met the requirements for vitamins B2, B6 and B12. The menus generated by ChatGPT and Copilot also generally met the requirements for vitamins A, B1 and B9. Furthermore, the Copilot-generated menus mostly met the requirements for vitamins E and C, whilst the menus generated by ChatGPT also mostly met the requirements for vitamin PP. The menus generated by Gemini very often failed to meet the requirements for specific vitamins, regardless of the energy value of the generated diet. None of the language models generated a single meal plan that met the requirement for vitamin D.

Overall, the meal plans generated by Gemini provided significantly less vitamins A, B1, B2 and B9 compared to the other models (*p* < 0.05, Kruskal–Wallis test), as well as significantly less vitamins D, E and C compared to the Copilot meal plans (*p* < 0.05, Kruskal–Wallis test) and significantly lower levels of vitamins B6 and B12 compared with ChatGPT (*p* < 0.05, Kruskal–Wallis test). However, the meal plans generated by Gemini contained significantly more vitamin E compared with those generated by ChatGPT (*p* < 0.001, Kruskal–Wallis test). Furthermore, the meal plans generated by ChatGPT contained significantly more vitamins B6 and B12 compared to those generated by Copilot but significantly less vitamins E, B9 and C (*p* < 0.05, Kruskal–Wallis test). Apart from these, no other statistically significant differences were found between the individual LLMs (*p* > 0.05, Kruskal–Wallis test).

## 4. Discussion

The aim of this study was to determine whether meal plans generated by the most popular language models—ChatGPT, Gemini and Copilot—would contain appropriate energy values and adequate amounts of nutrients. This is the first study of its kind to analyse such a large set of meal plans. It is also the first study to quantitatively assess the nutritional value of diets in the context of a specific country, taking into account local dietary standards and recommendations and economic conditions, as well as important aspects such as the issue of a balanced diet and environmental considerations. It is also the first paper from the 3(LM)Diet study, which comprehensively assesses the ability of language models to generate diets for the average user, as well as the associated risks.

### 4.1. The Energy Value of Generated Menus

The meal plans were almost always generated with a dietary energy value that was too low and significantly lower than the target value. The discrepancy between the recommended value and the actual ration generated was, in some cases, greater than 500 kcal; ChatGPT performed best with the lowest value (2000 kcal), Copilot with the intermediate value (2500 kcal), and Gemini with the highest value (3000 kcal). Overall, Gemini performed significantly worse compared to the other models. Depending on the generated energy value, as many as 62.9–94.3% of the meal plans fell outside the 10% margin of error. This huge disparity supports the conclusion that the generated energy value is far too low. Furthermore, it should be emphasised that, if an average user were to follow such a diet, preparing all the meal plans would not necessarily mean consuming the entire amount. Under-eating, throwing away leftovers and food waste may mean that actual energy intake, based on the generated diets, would be even lower, thereby exacerbating an already negative energy balance. The correct energy value of a diet is crucial for it to be properly balanced. Firstly, an appropriate energy value ensures the provision of all essential nutrients, including proteins, fats, and carbohydrates, as well as vitamins and minerals. Secondly, it effectively regulates body weight [47]. Ensuring the correct energy value is therefore crucial for an optimal and healthy diet. A significant proportion of the LLMs failed to meet this requirement.

Aslan & Sozlu (2025) reached the opposite conclusion. In their study, they presented a value that was close to the expected value [36]. However, these studies were based on one-day meal plans. In the present study, the individual days of the generated diets sometimes met, and even exceeded, the expected energy intake. It is possible that, when generating longer-term plans, the aforementioned authors would also have identified inaccuracies. Results consistent with this study were, in turn, obtained by Bayram et al. (2025) in their paper, in which 5-day meal plans generated by ChatGPT had a lower energy value compared to national recommendations [37]. These authors, however, also took into account the short duration of the generated diet and used only one LLM. Finally, in the 10-day plans generated by the same models as in this study, Kaçar et al. (2025) also observed discrepancies between the desired and generated values [38]. It is not possible, though, to determine whether, as in this study, these values were lower, as the authors only indicate the percentage difference between the generated and expected menus. These results are, however, more optimistic for ChatGPT and Copilot compared to the results obtained in this study. The energy values obtained in the studies by Kaçar et al. (2025) differed from the expected values by less than 10% for ChatGPT in 70% of meal plans and for Copilot in 50% of meal plans. The Gemini model, in turn, performed comparably to this study, showing significant discrepancies between the expected and generated energy values for low-energy diets, with only 2 out of 10 meal plans deviating by less than 10%. The discrepancies observed may also stem from the small number of meal plans generated in the study by Kaçar et al. (2025). On the one hand, this was insufficient to achieve comparable values for the two language models; on the other hand, it was sufficient to achieve similar results for Gemini. However, the discrepancies observed in the literature, as well as the low values obtained in this study, certainly point to the need for further analysis regarding the energy content of diets generated by LLMs.

### 4.2. Protein, Fat and Carbohydrate Content

The low energy value is due to an insufficient supply of individual macronutrients. When analysing the protein, fat and carbohydrate content, it should be noted that all models provided over 20% of their energy value from protein, with ChatGPT having the highest percentage. Although this percentage appears significant at first glance, it must be considered in the context of the total energy value. This was, in fact, too low. It may therefore mean that the amount of protein provided in the meal plans would be adequate if the negative energy balance in the diets observed were to be offset by other macronutrients, such as fats. The percentage of protein in the energy value would then decrease and would likely fall within acceptable limits. The percentage of energy derived from fats ranged from 20.4 ± 4.2% (ChatGPT 3000 kcal diet) to 29.6 ± 4.0% (Gemini 3000 kcal diet). Regardless of the LLM used or the total energy content, the proportion of energy derived from fat was too low. The current Polish nutritional standards for adults specify a Reference Intake of 30–40% of the total energy value [43]. Fats play an important role in the diet. They are a source of fat-soluble vitamins, as well as fatty acids, including polyunsaturated fatty acids, which are important for health [48,49,50]. Once absorbed by the body, they serve as a source of energy as well as provide structural support for many tissues, protection and stabilisation for organs, and regulation of specific processes [48,49,50,51]. An adequate intake of fat is therefore crucial for health, whilst an insufficient intake may impair certain functions and lead to deficiencies. The carbohydrate intake recommended in Polish dietary guidelines is 45–65% of the diet’s energy value. It can therefore be concluded that all models, across all energy variations, fell within the recommended range. A negative energy balance is therefore due to an insufficient intake of fat. This may be because certain models frequently omitted fats used in cooking. Although in most cases these fats were included in the generated meal plans, the omission of such additions in some instances may have resulted in a lower-than-recommended fat intake and, consequently, a lower-than-expected energy value. Including additional fats in sandwiches or with certain dishes could therefore balance the energy value and improve the macronutrient ratios, which are already at an acceptable level.

It is worth noting that, as with the reference values set out in Polish standards, the generated meal plan also shows the percentage contribution of macronutrients to the energy content of the diet, which is partly inconsistent with the World Health Organisation’s (WHO) guidelines on a healthy diet [14]. This is because, although these guidelines specify that carbohydrates should account for 45–75 per cent of the diet’s energy value and that fat should account for 15–30 per cent of the diet’s energy value—which is consistent with the generated meal plan—the proportion of protein exceeds the recommended range of 10–15 per cent of the diet’s energy value [14]. It should be noted, however, that the meal plans have an energy value that is too low compared to the target value. It cannot, therefore, be ruled out that, should the language models add foods rich only in carbohydrates and fat in order to balance the energy content of the diet, these proportions would be brought into balance. Notwithstanding the above, these values remain incorrect in relation to the specified requirement that the generated diet should comply with the recommendations set out in the conditions of a specific country.

Logan et al. (2025) reached the opposite conclusions in their research, demonstrating that the macronutrient content of the generated meal plans was consistent with the guidelines and adapted to cultural constraints. However, this research was conducted in the specific context of cancer care. Furthermore, the authors used complex prompts across various categories, rather than simple commands simulating an average user [52]. In studies involving simulated diabetic patients, Bayram et al. (2025), in turn, demonstrated an overestimation of fat content in the case of ChatGPT, as well as an underestimation of carbohydrates in the case of DeepSeek [53]. Although language models generally cope well with providing appropriate intake of macronutrients, they have certain shortcomings that prevent them from accurately estimating all macronutrients.

### 4.3. Minerals and Vitamins

Finally, attention should be paid to vitamins and minerals. It should be emphasised that, for the most part, the language models generated meal plans with adequate levels of minerals and vitamins—mainly from the B group—although individual models often outperformed others in terms of specific minerals and vitamins. Gemini undoubtedly performed relatively poorly in terms of these nutrients, particularly with lower-calorie meal plans, often providing insufficient amounts of both vitamins and minerals. Furthermore, ChatGPT struggled to ensure adequate levels of vitamin E, and none of the models provided sufficient levels of vitamin D on any of the days for which meal plans were generated.

Vitamin E is an antioxidant that performs many functions in the body, including the regulation of inflammatory processes, immune function, gene expression and cognitive function [54]. Vitamin D, on the other hand, although mainly known and associated with bone health and calcium metabolism, also plays a role in other systems, and its deficiency is linked to cardiovascular, respiratory and gastrointestinal conditions; neurological and metabolic disorders; cancers; and skin diseases [55]. It is worth noting, however, that the main source of vitamin D is UVB radiation and the resulting synthesis in the skin [55,56]. To address nutritional deficiencies, which are very commonly observed in studies, mandatory vitamin D supplementation is recommended for countries with limited exposure to sunlight, including Poland [57,58]. It can therefore be concluded that, apart from vitamin E in the case of ChatGPT, both Copilot and ChatGPT generated meal plans with generally adequate levels of vitamins and minerals. Unfortunately, Gemini failed to meet these criteria in most cases, and the dietary plan generated by this model poses a clear risk of nutritional deficiencies. An uncritical approach to using such a model may pose a serious risk to the individual using it.

None of the diets generated by any of the models exceeded the upper tolerable intake level. Although the vitamin A content in the generated diets was high and often exceeded the amounts specified in the standards, it did not reach a level at which it could cause toxic effects [43].

It should be noted that excessive sodium intake has been linked to a number of adverse health effects, such as high blood pressure and certain types of cancer [10,59,60,61,62]. However, it should be noted that Polish standards have not established a UL value for sodium [43]. Similarly, EFSA experts concluded that there is insufficient evidence to establish a UL for this nutrient [63,64]. Consequently, a risk assessment relating to sodium intake was beyond the scope of this study and will be addressed in a separate paper.

### 4.4. Strengths and Limitations

This study has both strengths and weaknesses. One of its valuable aspects is the large number of meal plans, which is unprecedented in similar studies. This allows for a broader perspective on the long-term use of diets generated by LLMs, as well as a comparison of nutritional values across models. Another advantage is the use of three LLMs that are popular and widely used in modern society. This enhances the study’s practical implications in terms of the likelihood of such diets being adopted by the public, as well as the potential consequences that may follow. Finally, a strength of this study is that it is set within the context of a specific country, as well as that of a potential user who lacks the necessary knowledge to verify the information obtained. The study, therefore, has practical implications, demonstrating the opportunities and risks present in contemporary communities.

However, this study also has certain limitations. The main limitation is the use of non-expert prompt formulation. On the one hand, refining the queries more precisely, as well as providing input data to the tools, could improve the accuracy of the generated meal plans and ensure greater compliance with nutritional standards. On the other hand, this study simulates an average user with no knowledge of how to formulate queries correctly or of nutrition-related information. It is, therefore, a study that is more grounded in real-world conditions. Consequently, these results should be interpreted as reflecting the performance of LLMs under conditions typical of the average user, rather than expert-optimised conditions. Another limitation of the study is the use of only three language models. A larger number of LLMs would certainly provide a broader picture of AI’s ability to generate diets. Moreover, LLMs are frequently updated, and results obtained with specific versions or access conditions may not be stable over time. However, a large sample of meal plans with different energy levels was utilised across the most popular tools. Whilst it is possible to expand this set, the picture provided is sufficient for the context of the study—the real opportunities and risks for the average user on the days of the assessment. A further limitation is the absence of any indication of gender or nationality in the prompt. Although the nutritional value has been analysed with regard to both women and men following the diet, certain preferences or dietary choices are dictated by gender or cultural context. A prompt containing such information could generate different results. Ultimately, the software itself is a limitation of the study. Although professional dietary software was used, it provides only an estimate of the nutritional value of the meal plans. Such software is based on certain average values for product data categories. By considering the relevant products available on the market and carrying out laboratory analyses, different results might be obtained. These limitations should, however, be considered when interpreting the results of this study.

## 5. Conclusions

The results of this study indicate that meal plans generated by LLMs have shortcomings. The meal plans presented were characterised by an excessively low energy value, which was the result of an insufficient amount of macronutrients, most likely fat. Although the protein content appears to be too high, offsetting the negative energy balance with the other macronutrients may reduce this defect. The diets generated by ChatGPT and Copilot generally provided adequate amounts of vitamins and minerals, although individual days of meal plans did not meet the amounts specified in the standards. The diets generated by Gemini, particularly those with lower energy content, contained insufficient amounts of micronutrients, especially vitamins. However, research on this topic is extremely scarce. Further research is needed on various LLMs across different scenarios and patient simulations to determine the models’ suitability for generating diets. However, unsupervised use of LLM-generated diets by non-expert users may produce nutritionally inaccurate menus and should not replace professional dietary counselling.

## Figures and Tables

**Table 1:** Prompts given to the LLM for generating menus.

Prompts	ChatGPT	Gemini	Copilot
General prompt	I would like you to create a 35-day (5-week) meal plan for me. The meal plan should be based on ……… kcal ^1^ and be designed for an average healthy person aged 19–30 with a normal body weight within the standard range. The meal plan should be based on a model for people who eat all types of food; it does not need to exclude any food products. I would like this diet to be balanced each week, so that I can swap weeks around, as well as across the entire period. I would like the meal plan to meet the following criteria: 1. It complies with Polish dietary guidelines, 2. It meets the nutritional requirements for the relevant age group as set out in Polish human nutrition standards, 3. The cost of the menu is acceptable and falls within the range of 110 PLN per week, 4. It should be consistent with the principles of a sustainable diet and be environmentally friendly, 5. The dishes included in the menu should be possible for someone with average cooking skills to prepare, without the need for elaborate culinary techniques, 6. It should specify the weights of the products used in the menu.		
Additional prompt			
Generating additional days	✓	✓	x
Improvements on individual days	✓	✓	x
Clarification of weights	✓	✓	x
Clarification of the ingredients of dishes	✓	x	x

^1^ The target calorie intake varied across the three meal plan generation commands: 2000 kcal, 2500 kcal, and 3000 kcal.

**Table 2:** Energy value of the generated diets.

Expected Energy Value	Actual Energy Value					
	Mean ± SD	Median	Min–Max	95%CI	T/W ^	Mean/Median Difference Between the Expected and the Observed Value
	CHATGPT					
2000 kcal	1711 ± 169 ***	1701	1407–2025	1652–1769	−10.1	289 ± 169 a
2500 kcal	2152 ± 191 ***	2159	1821–2663	2086–2217	−10.8	348 ± 191 cd
3000 kcal	2522 ± 212 ***	2585	2001–2895	2449–2594	−13.3	478 ± 212
	GEMINI					
2000 kcal	1511 ± 142	1540 ***	1103–1689	1463–1560	−5.2	460 ab
2500 kcal	1965 ± 184 ***	1968	1605–2359	1902–2028	−17.2	535 ± 184 ce
3000 kcal	2629 ± 258 ***	2617	2148–3054	2560–2717	−8.5	371 ± 258 f
	COPILOT					
2000 kcal	1758 ± 175	1699 ***	1527–2221	1698–1819	−4.7	301 b
2500 kcal	2212 ± 153	2216 ***	2012–2579	2159–2264	−5.1	284 de
3000 kcal	2515 ± 243	2454 ***	2202–3060	2432–2599	−5.1	546 f

* *p* < 0.05; ** *p* < 0.01; *** *p* < 0.001. ^ The one-sample *t*-test for variables with a distribution approximating a normal distribution and mean ± SD was taken into account, and the one-sample Wilcoxon test for variables with a non-normal distribution and medians was taken into account. a, b, c…—values denoted by the same letters differ significantly from one another (Kruskal–Wallis test; post hoc analysis used the Dunn test with Bonferroni correction).

**Table 3:** Percentage contribution of protein, fat and carbohydrates to the energy value of the generated diets.

Expected Energy Value	ChatGPT [% of Energy Value]			Gemini [% of Energy Value]			Copilot [% of Energy Value]		
	Protein	Fat	CHO	Protein	Fat	CHO	Protein	Fat	CHO
2000 kcal	22.8 ± 2.7	23.5 ± 3.8	57.2 ± 4.3	22.2 ± 3.8	27.3 ± 3.0	52.7 ± 5.0	20.6 ± 2.3	25.2 ± 4.5	57.3 ± 3.8
2500 kcal	22.4 ± 2.0	21.3 ± 3.3	59.8 ± 3.8	20.3 ± 2.8	27.3 ± 3.2	54.8 ± 3.7	19.5 ± 1.9	25.5 ± 4.4	55.8 ± 3.9
3000 kcal	21.7 ± 2.7	20.4 ± 4.2	60.7 ± 5.2	19.2 ± 2.9	29.6 ± 4.0	53.8 ± 5.0	19.9 ± 1.8	26.1 ± 3.9	57.1 ± 3.4
OVERALL	22.3 ± 2.5	21.8 ± 4.0	59.3 ± 4.6	20.6 ± 3.4	28.1 ± 3.6	53.8 ± 4.7	20.2 ± 2.0	26.1 ± 4.3	56.8 ± 3.8

The table shows the mean ± SD. OVERALL row present data across the three energy targets. The values for individual rows exceed 100% due to the programme’s calculation taking into account the energy value derived from dietary fibre (2 kcal/g of fibre).

**Table 4:** Mineral content in the generated diets.

Mineral	Unit	ChatGPT			Gemini			Copilot			Standard EAR/AI
		2000 kcal	2500 kcal	3000 kcal	2000 kcal	2500 kcal	3000 kcal	2000 kcal	2500 kcal	3000 kcal	
Sodium	g	2.55 ± 0.8	2.94 ± 0.9	2.79 ± 0.7	1.8 ± 0.7	2.16 ± 0.8	3.06 ± 0.9	2.07	2.67	2.90	1.5
	%	5.7	2.9	0	37.1	17.1	0	25.7	8.6	8.6	
Potassium	g	3.74 ± 0.6	4.55 ± 0.6	4.69 ± 0.8	2.87	3.12 ± 0.5	4.24 ± 0.7	3.23	3.73	4.34	3.5
	%	42.9	5.7	8.6	85.7	80	8.6	60	28.6	0	
Calcium	mg	907	1051 ± 233	1177 ± 247	786	685 ± 234	1069 ± 264	851 ± 201	944	1100 ± 238	800
	%	37.1	17.1	11.4	57.1	65.7	17.1	45.7	14.3	14.3	
Phosphorus	mg	1754	2256 ± 312	2457 ± 464	1447	1680 ± 287	2303 ± 353	1625 ± 294	2031	2343	580
	%	0	0	0	0	0	0	0	0	0	
Magnesium	mg	396	506 ± 121	540 ± 123	312 ± 73	388 ± 94	519	358	473	518	255/330 ^
	%	0/5.7	0/2.8	0/2.8	20/62.9	5.7/20	0/0	14.9/34.3	0/17.1	0/8.6	
Iron	mg	13.9	16.4 ± 2.9	17.9 ± 3.4	9.6 ± 3.4	12.9 ± 2.4	19.3 ± 5.5	13.2	16.4	18.8	8/6 ^
	%	0/0	0/0	0/0	20/0	0/0	0/0	0/0	0/0	0/0	
Zinc	mg	13.3 ± 1.6	16.4	17.1	9.6 ± 1.5	12.3 ± 1.9	17.3	11.8	14.9	16.6	6.8/9.4 ^
	%	0/0	0/0	0/0	2.9/48.6	0/5.7	0/0	0/0	0/0	0/0	
Copper	mg	1.52 ± 0.2	1.79 ± 0.3	1.96	1.18 ± 0.3	1.57 ± 0.4	2.29 ± 0.6	1.48	1.75	2.08	0.7
	%	0	0	0	2.9	0	0	0	0	0	
Manganese	mg	7.67 ± 1.5	9.71	9.14 ± 2.2	3.78	5.03	8.60 ± 3.6	5.27	6.53	8.09	1.8/2.3 ^
	%	0/0	0/0	0/0	14.3/28.6	0/2.9	0/0	0/0	0/0	0/0	
Iodine	µg	121	139	125 ± 37	70.2	73.3	104.7	64.5	70.5	81.7	95
	%	17.1	14.3	28.6	65.7	51.4	54.9	54.9	57.1	57.1	

Where the distribution is close to normal, the mean ± SD is given; where the distribution deviates from normal, the medians are given. The EAR standard was selected as the default. Where no EAR standard had been specified, the AI standard was applied. %—percentage of days on which the standard was not met; ^—for women and men, respectively.

**Table 5:** Vitamin content in the generated diets.

Vitamin	Unit	ChatGPT			Gemini			Copilot			Standard EAR/AI
		2000 kcal	2500 kcal	3000 kcal	2000 kcal	2500 kcal	3000 kcal	2000 kcal	2500 kcal	3000 kcal	
A	µg	1750 ± 606	2019	1423	812	803	1299	1751	2119	2179	500/630 ^
	%	2.9/5.7	2.9/14.9	2.9/5.7	14.3/22.9	8.6/20	0/0	2.9/2.9	2.9/2.9	0/2.9	
D	µg	2.91 ± 1.9	3.95 ± 2.4	3.03	2.52 ± 1.4	2.60	2.48	3.0 ± 1.7	3.38	3.40	15
	%	100	100	100	100	100	100	100	100	100	
E	mg	9.45 ± 2.4	10.1 ± 2.5	9.06	9.60 ± 3.0	12.12	19.14	12.03	16.28	17.47 ± 3.3	8/10 ^
	%	28.6/51.4	20/48.6	28.6/65.7	34.3/51.4	34.3/34.3	0/5.7	17.1/20	0/5.7	0/0	
B1	mg	1.37 ± 0.3	1.65	1.95 ± 0.5	1.0	1.29	2.02 ± 0.6	1.32	1.58	1.96	0.9/1.1 ^
	%	2.9/14.3	0/2.9	0/0	37.1/65.7	8.6/25.7	0/8.6	0/0	0/0	0/0	
B2	mg	2.31 ± 0.3	2.74 ± 0.4	3.18 ± 0.4	2.12 ± 0.3	2.11 ± 0.5	2.76 ± 0.5	2.26	2.62 ± 0.4	3.1	0.9/1.1 ^
	%	0/0	0/0	0/0	0/0	0/0	0/0	0/0	0/0	0/0	
PP	mg	14.1	19.2	16.4	13.7 ± 5.8	23.0	23.8	15.1	17.5	20.2	11/12 ^
	%	11.4/20	5.7/5.7	5.7/11.4	34.9/42.9	28.6/28.6	0/8.6	37.1/40	28.6/37.1	2.9/14.3	
B6	mg	2.38 ± 0.4	2.78 ± 0.5	2.95 ± 0.6	1.82 ± 0.3	2.26	3.09	2.12 ± 0.5	2.42	2.68	1.1
	%	0	0	0	2.9	0	0	0	0	0	
B9	µg	393 ± 57	469 ± 80	535 ± 95	297 ± 49	356	469	429	506	593	320
	%	8.6	0	0	62.9	25.7	2.9	0	0	0	
B12	µg	4.95 ± 1.1	6.07	6.62 ± 1.6	5.06	4.77	5.55	4.14	5.20	5.49	2.0
	%	0	0	0	0	0	0	0	0	0	
C	mg	104.3	107.2	80.1	66.0	85.2	108.8	148.5	175.3	212.9	60/75 ^
	%	25.7/42.9	8.6/25.7	14.3/31.4	37.1/60	40/42.9	2.9/20	0/17.1	0/17.1	0/0	

Where the distribution is close to normal, the mean ± SD is given; where the distribution deviates from normal, the medians are given. The EAR standard was selected as the default. Where no EAR standard had been specified, the AI standard was applied. %—percentage of days on which the standard was not met; ^—for women and men, respectively.

## Data Availability

The data presented in this study are openly available in RepOD at https://doi.org/10.18150/9GVUEG.

## Abbreviations

The following abbreviations are used in this manuscript:

LLM	Large Language Model
AI	Artificial Intelligence
